# Rare presentation of chronic recurrent multifocal osteomyelitis of the Iliac wing mimicking Ewing’s sarcoma

**DOI:** 10.4102/sajr.v25i1.2030

**Published:** 2021-02-18

**Authors:** Pankaj Nepal, Syed I. Alam, Sadia Sajid, Joshua Sapire, Vijayanadh Ojili

**Affiliations:** 1 Department of Radiology, Faculty of Sciences, St. Vincent’s Medical Center, Bridgeport, United States of America; 2 Department of clinical imaging, Faculty of Sciences, Hamad medical corporation, Doha, Qatar; 3 Department of Radiology, University of Texas Health, San Antonio, Texas, United States of America

**Keywords:** Chronic recurrent multifocal osteomyelitis, CRMO, MRI, Ilium, rare

## Abstract

This report describes a case of chronic recurrent multifocal osteomyelitis (CRMO) in an 11-year-old girl, involving the iliac bone as an initial, solitary site. Atypical imaging features were suspicious of a bone tumour, such as Ewing’s sarcoma. Chronic recurrent multifocal osteomyelitis is a great masquerader and can present atypically. Radiologists should be familiar with both typical and atypical presentations, to determine an accurate diagnosis and guide appropriate management. Timely diagnosis may avoid invasive bone biopsy and inappropriate long-term antibiotic prescription for children.

## Introduction

Chronic recurrent multifocal osteomyelitis (CRMO) is a rare idiopathic inflammatory skeletal disorder, which is also known as chronic non-bacterial osteomyelitis. It commonly affects children and adolescents with predominant involvement of the metaphysis of long bones. Several cases have been reported worldwide affecting every age group and almost all the bones.^1^ Chronic recurrent multifocal osteomyelitis is a diagnosis of exclusion, and atypical cases demand additional investigations to exclude major differentials, such as infection and bone tumours. Magnetic resonance imaging (MRI) is the modality of choice for investigation, and the diagnosis is based on combined clinical and imaging follow-up to demonstrate relapsing and remitting episodes.^2^

## Case report

An 11-year-old girl presented to the orthopaedic clinic with relapsing and remitting chronic right hip pain for 2 years, especially at night. This gradually led to limitation in her daily activities. There were no associated symptoms of weakness, numbness or paraesthesia. Her medical history was unremarkable. She denied any trauma, fever or swelling. Laboratory work-up was normal except for an elevated erythrocyte sedimentation rate (ESR) (55 mm/h). Leucocytosis was characteristically absent. On physical examination, she had localised tenderness over the right iliac wing. Hip flexion, extension and other ranges of motion were unremarkable.

**FIGURE 1:**
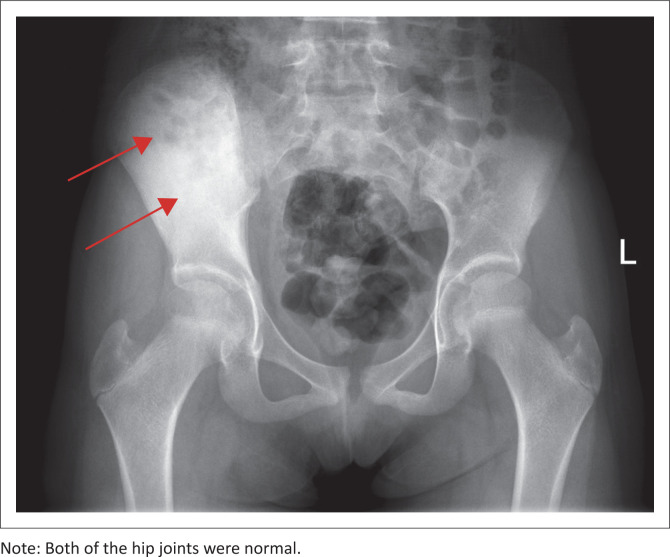
An 11-year-old-girl with chronic recurrent multifocal osteomyelitis: Initial plain radiograph of the pelvis demonstrated diffuse sclerosis of the right iliac wing (arrows).

**FIGURE 2:**
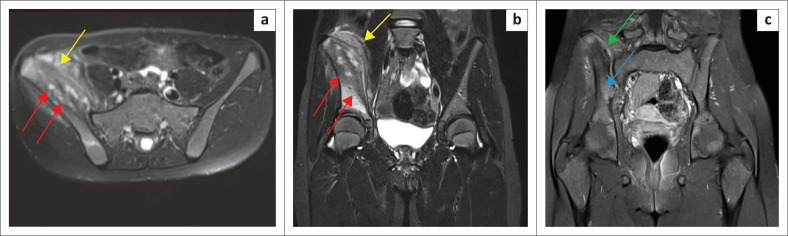
Magnetic resonance imaging of the pelvis performed without and with gadolinium: (a) Axial T2 fat-saturated image of the pelvis showing heterogeneous marrow signal within the right iliac wing with marrow expansion (red arrows). Diffuse layered periosteal reaction was noted with increased T2 signal in the adjacent part of the iliacus and gluteal muscles (yellow arrow). (b) Coronal short tau inversion recovery (STIR) image of the pelvis showing layers of periosteal reaction (yellow arrow). Increased marrow signal could be seen extending close to the acetabular roof but without intra-articular extension (red arrows). (c) Coronal fat-saturated image gadolinium enhanced T1 weighted image indicated mild sacroiliitis with post contrast enhancement (green arrow) and heterogeneous marrow enhancement (blue arrow). In addition, there was enhancement of the adjacent muscles.

Initial imaging included a plain radiograph of the pelvis including both hip joints (Figure 1). The right iliac wing demonstrated diffuse sclerosis and the hip joints were normal. Magnetic resonance imaging of the pelvis was then performed both without and with gadolinium contrast (Figure 2). Heterogeneous marrow signal was seen within the iliac wing with marrow expansion. Diffuse periosteal reaction was noted with increased T2 signal in the adjacent part of the iliacus and gluteal muscles. Layers of periosteal enhancement was evident with enhancement of adjacent involved muscles. The mass could be seen extending close to the acetabular roof, but no intra-articular extension was noted. Mild sacroiliitis was present with post-contrast enhancement. The imaging findings were suspicious for a bone tumour such as Ewing’s sarcoma, considering the patient’s age. Absence of fever, leucocytosis and negative blood cultures were clinically less suspicious for osteomyelitis. Biopsy of the iliac bone was performed, which showed reactive bone formation with chronic inflammation. Specimens were negative for malignancy and infection. The patient was managed conservatively with anti-inflammatory therapy and local treatment.

**FIGURE 3:**
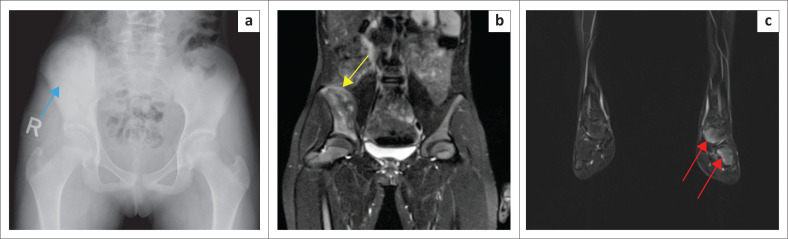
Follow-up imaging with a radiograph of the pelvis and whole-body magnetic resonance imaging (MRI) performed 3 months after treatment: (a) Plain radiograph of the pelvis showing similar findings of sclerosis of right iliac wing. (b) Magnified fat-saturated T2 weighted coronal image on whole-body MRI shows significant improvement of the oedema and surrounding periosteal reaction (arrow). Note the absence of adjacent muscle inflammation. (c) Magnified section of the whole-body MRI, coronal T2 weighted fat-saturated MR image of the left lower leg showing increased marrow signal involving the inferior talus, cuboid (arrows) and base of 5th metatarsal bone (not shown). This involvement of multiple bones with regression of inflammation in the ilium after conservative treatment confirms the diagnosis of chronic recurrent multifocal osteomyelitis.

Follow-up imaging with a pelvic radiograph and whole-body MRI were performed 3 months after treatment (Figure 3). There was redemonstration of the heterogeneous marrow signal involving the iliac bone, which showed improvement. The periosteal thickening and reaction were also improved. Abnormal T2 hyperintensity of the adjacent gluteus and iliacus muscles appeared significantly regressed. There was increased T2 marrow signal involving the inferior talus, cuboid and base of 5th metatarsal bones. This involvement of multiple bones with regression of inflammation in the ilium confirmed the diagnosis of CRMO.

## Discussion

Chronic recurrent multifocal osteomyelitis is an idiopathic inflammatory disease, mostly prevalent in children and adolescents, that results in episodic bone pain.^3,4,5,6^ It is an uncommon condition with an estimated prevalence of one in a million.^6^ Interestingly, for some unknown reasons, literature indicates that up to 85% of the affected are females.^6^ Chronic recurrent multifocal osteomyelitis is also known as chronic non-bacterial osteomyelitis as no pathogens grow on blood culture and antibiotics do not relieve the symptoms.^6,7^ The aetiology remains elusive but has been associated with many dermatological and autoimmune disorders such as psoriasis, Takayasu’s arteritis, Crohn’s disease, ulcerative colitis, SAPHO (synovitis, acne, pustulosis, hyperostosis, osteitis) syndrome and some genetic disorders.^8,9,10^ Clinically, patients present with non-specific symptoms of pain, swelling and limited range of motion. Systemic symptoms such as fever, chills and lethargy are unusual.^2^

Chronic recurrent multifocal osteomyelitis lesions are often multifocal with typical involvement of the distal metaphysis of the long bones, which undergo sclerosis or hyperostosis over time. Common skeletal sites include long tubular bones and the clavicle, but cases have been described throughout the skeleton including the spine, pelvis, sternum, scapula, mandible, hands and feet.^2^ The lower extremity is involved three times more frequently than the upper extremity, with the most common bone involvement being the tibia. A study by Andronikou et al.^11^ described the pattern as multifocal with predominantly tibial involvement or pauci-focal clavicular and spinal disease. The condition typically affects the metaphysis more commonly, with epiphyseal involvement in a third of the cases.^11^ Although the disease is multifocal, the condition may present with involvement at a single site. Thus, further evaluation of the entire skeleton is indicated to identify multifocal lesions that may be clinically silent.^2^ Whole-body imaging may be performed both with 99mTc bone scintigraphy or whole-body MRI, each with their own limitations, considering cost versus radiation.

Involvement of the ilium as a potential site for CRMO has been reported infrequently.^2,6,12,13^ Chronic recurrent multifocal osteomyelitis of the pelvic bone itself is rare and the site of predilection in pelvis includes metaphyseal equivalents such as the ischiopubic synchondrosis and sacroiliac joints.^2,14,15^ Pelvic CRMO can rarely manifest as sclerosis of the iliac wings.^2,6^

There is considerable overlap in the imaging findings, which are not pathognomonic. Metaphyseal involvement without periosteal reaction is the typical presentation of CRMO.^6^ Significant periosteal reaction and adjacent soft tissue component, as in our case, delayed the diagnosis as the initial impression was either Ewing’s sarcoma or Langerhans cell Histiocytosis. Osteomyelitis was low in clinical suspicion because of the absence of systemic findings, leucocytosis and negative blood culture. The unusual location of the initial solitary lesion in the right iliac wing in our case added to difficulties in the diagnosis of CRMO.

Although a prolonged and fluctuating relapsing-remitting course is pathognomonic, the typical history may be lacking unless sought for or absent as in our case. A team approach of clinicians, orthopaedic surgeons, radiologists and pathologists is crucial in reaching the diagnosis such as in our case.

As a result of the overlapping imaging features, atypical cases are often biopsied. The biopsy may show inflammation and absence of culture of causative organisms. It will also help in excluding bone tumours. Inflammatory disease with absence of a positive culture is a clue for CRMO if this differential is in mind, otherwise it may be mismanaged with prolonged antibiotics.

Few points to consider from our case are: (1) chronic 2-year history of bone pain (2) diffuse sclerosis of the iliac bone on plain radiography (3) involvement of the sacroiliac joint with features of sacroiliitis (4) multifocal involvement of the talus, cuboid and metatarsal bones and (5) interval improvement of inflammation involving the bone marrow and adjacent muscles with anti-inflammatory treatment. A recent update in the treatment approach recommends using either bisphosphonate or biological therapy in addition to the anti-inflammatory and local treatment.^1,13^

## Conclusion

Chronic recurrent multifocal osteomyelitis as a solitary lesion at an uncommon site may masquerade as various forms of infection or bone tumours. The diagnosis depends on a multidisciplinary approach; thus, the radiologist must be familiar with this diagnosis and should recommend additional whole-body imaging to aid in diagnosis, if suspected.
